# Unfavorable Prognostic Effects of the Stem Cell Pluripotency Factor Sox2 in Feline Invasive Mammary Carcinomas

**DOI:** 10.3389/fvets.2020.622019

**Published:** 2021-01-22

**Authors:** Yohan Truchot, Elie Dagher, Jérôme Abadie, Frédérique Nguyen

**Affiliations:** ^1^AMaROC (Animal Cancers, Models for Research in Comparative Oncology), Oniris, Nantes Atlantic College of Veterinary Medicine, Food Science and Engineering, Nantes, France; ^2^Université de Nantes, Inserm, CRCINA, Nantes, France; ^3^Integrated Center for Oncology Nantes/Angers, Nantes, France

**Keywords:** androgen receptor, cat, mammary carcinoma, prognosis, regulatory T cells, SOX2, survival

## Abstract

**Background:** Sex-determining Region Y (SRY)-box transcription factor-2 (Sox2) belongs to the “Yamanaka's factors,” necessary and sufficient to convert somatic cells into pluripotent stem cells. In breast cancers, Sox2 expression has been associated with poor prognosis, and resistance to therapy. The aims of this study were to determine the frequency of Sox2 positivity in feline invasive mammary carcinomas (FMCs), its relationships with other clinical-pathologic variables, and with patient outcomes.

**Materials and Methods:** This study relies on a previously described retrospective cohort of 180 FMCs, diagnosed in female cats treated by mastectomy alone, with 2-year follow-up. Sox2 (clone SP76), Estrogen Receptor alpha (ER), Progesterone Receptor (PR), Ki-67, Human Epidermal growth factor Receptor 2 (HER2), Androgen Receptor (AR), Bcl-2, Forkhead box protein A1 (FOXA1), basal markers and FoxP3-positive regulatory T cells (Tregs) were detected by automated immunohistochemistry. Sox2 expression was quantitated as an index (percentage of neoplastic cells demonstrating a positive nuclear signal). The FMCs were considered Sox2-positive at threshold >42%.

**Results:** Sox2 was not expressed in the normal mammary gland or in mammary hyperplasia without atypia, but was occasionally detected in atypical hyperplasia. In FMCs, the mean Sox2 index was 38 ± 30%, and 79/180 FMCs (44%) were Sox2-positive. Sox2 expression was associated with older age at diagnosis, lymphovascular invasion, high Ki-67 proliferation indexes, low PR and FOXA1 expression, and increased numbers of tumor-associated Tregs, but was not significantly associated with the clinical stage, histological types, and histological grade. By multivariate survival analysis, Sox2 was associated with poor cancer-specific survival (Hazard Ratio = 1.48, 95% confidence interval 1.04–2.11, *p* = 0.0292), independently of the pathologic tumor size, pathologic nodal stage, distant metastasis, and AR expression. A rare subgroup of FMCs characterized by an AR+Sox2–phenotype (19/180 cases, 11%) was associated with very favorable outcomes.

**Conclusion:** Sox2 expression was associated with poor cancer-specific survival of female cats with invasive mammary carcinomas, as previously reported in human breast cancer, but was more commonly expressed in cats than reported in breast cancers. Sox2 showed complementarity with AR in FMC prognostication.

## Introduction

The transcription factor Sex-determining Region Y (SRY)-box transcription factor-2 (Sox2), first sequenced in humans in 1994 (1), plays a critical role in maintenance of embryonic stem cells (2). In 2006–2007, Takahashi and Yamanaka demonstrated that adult somatic cells could be converted into induced pluripotent stem cells by transduction of only 4 transcription factors: Sox2, Oct4 (octamer-binding transcription factor 4), c-Myc and Klf4 (Kruppel-like factor 4), in mice (3) and humans (4). Thereafter, these four transcription factors were considered stem cell pluripotency factors and sometimes referred to as “Yamanaka's factors.”

Poorly differentiated human breast cancers tend to express an “embryonic stem cell-like” signature that contains activation targets of Nanog, Oct4, Sox2, and c-Myc (5), and it has been demonstrated in the MCF-7 breast cancer cell line that Sox2 up-regulates 145 genes, and down-regulates 41 genes (6). In breast cancer cell lines, Sox2 increases mammosphere formation (7), up-regulates *CCND1*, the cyclin D1-encoding gene, thus facilitating the G1/S transition of the cell cycle (6), activates the WNT signaling pathway and the epithelial-to-mesenchymal transition (8).

At threshold >0% for positivity, it is estimated that 9–33% of breast cancers are Sox2-positive (7, 9–13). Positivity to Sox2 has been associated with a larger tumor size (11–15), presence of lymph node metastasis (8, 9, 11, 15), a higher histological grade (8, 9, 14–16), a higher Ki-67 proliferation index (13, 14, 16), and negativity to Estrogen Receptor (ER) and Progesterone Receptor (PR) (12, 14). Accordingly, Sox2 positivity is rare in luminal-A (ER+, PR+, low Ki-67) breast cancers (16), and more common in triple-negative (ER–, PR–, HER2–) breast cancers (9, 12, 14, 15). Sox2-positive breast cancers have been associated with poor disease-free survival (10, 13, 14), and poor overall survival (13). Finally, Sox2 has been associated with tamoxifen resistance of hormone-dependent breast cancers (17), and paclitaxel resistance of triple-negative breast cancer cells (18).

Here, we hypothesized that Sox2 expression could be involved in the particularly aggressive clinical behavior of invasive mammary carcinomas in cats. The first objective of this study was to determine the frequency of Sox2 expression in feline mammary carcinomas, and its associations with clinical-pathologic features such as the clinical stage, histological grade, proliferation index, and hormone receptor expression. The second objective was to determine whether Sox2 was associated with patient outcomes.

## Materials and Methods

### Materials

For the study of Sox2 expression in normal tissues, samples from healthy cats dead from accidental causes were collected at necropsy in our facilities. For the study of Sox2 expression in feline mammary carcinomas, a previously described cohort of 180 female cats was used. These cats diagnosed with invasive mammary carcinoma were treated solely by mastectomy (19–21). Owners signed an informed consent for approval of inclusion of their pet cats in the present study. The local animal welfare committee of our institution (CERVO, Comité d'Ethique en Recherche clinique et épidémiologique Vétérinaire d'Oniris) approved the design of the study.

### Patient Information and Follow-Up

The clinical collected data were patient age at diagnosis, breed, weight, neuter status (intact or neutered female cats), reproductive history (parity, contraceptive exposure, age at ovariectomy if applicable), medical history (previous non-cancerous mammary lesions, intercurrent diseases), the clinical tumor size and clinical stage of the mammary carcinoma at diagnosis, according to Morris (22), a four-stage system adapted from Owen (23).

The minimal length of follow-up was 2 years. Veterinary practitioners and owners of the cats of the present study were asked to provide the dates of occurrence of any local recurrences, regional spread (nodal metastases), and distant metastases (confirmed by medical imaging or necropsy), as well as the date and cause of death. For locoregional recurrence risk analyses, censoring was “1” in case of true recurrence (at the same site as the mammary carcinoma included), new primary mammary tumor, or nodal metastasis, and “0” otherwise. The distant metastasis-free interval (DMFI) corresponded to the time period between diagnosis and distant metastasis confirmation. The disease-free interval (DFI), which is indicative of cancer progression, was censored “1” in case of locoregional recurrence and/or distant metastasis, and “0” in the absence of such events. Overall survival was censored “0” for patients still alive at the end of the follow-up period, and “1” for dead patients, whatever the cause of death. The censure applied for cancer-specific survival analyses was “1” if the animal had died from cancer, and “0” if the cat was still alive at the end of follow-up, or died from unknown cause, or died from a cause unrelated to the mammary carcinoma.

### Histopathological Methods

Although the cases originated from two different laboratories of veterinary diagnostic pathology, they were all centrally collected at our institution (Oniris, Nantes Atlantic College of Veterinary Medicine, Food Science and Engineering, France). The paraffin blocks were used to generate new Hematoxylin-Eosin-Saffron (HES)-stained slides, as well as the slides for immunohistochemical analyses. Three specialists in veterinary pathology (DVM, ECVP diplomates) and a MD specialist in breast cancer pathology analyzed the slides blinded to patient outcomes and reached consensus in each case.

The recorded histopathological parameters were: unifocality, multifocality (multiple carcinomas within the same mammary gland) or multicentricity (multiple carcinomas in different mammary glands), the pathologic tumor size (in millimeters, measured on HES slides), the pathologic nodal stage (pN0: absence of nodal metastasis, confirmed by negative pancytokeratin immunohistochemistry; pN+: presence of nodal metastases of any size, even isolated tumor cells; pNX: lymph node not available for histopathology), the histological type (tubular, papillary, tubulopapillary, cribriform, mucinous, solid, adenosquamous, squamous cell, anaplastic), the histological grade according to Elston and Ellis (24, 25), the mitotic-modified Elston and Ellis (MMEE) grading system (24, 26), the novel grading system (NGS) for FMCs (24, 26), presence/absence of lymphovascular invasion, dermal infiltration, muscle infiltration, squamous differentiation, central necrosis (of any type and any extent), margin status (negative: tumor-free vs. positive: infiltrated), and tumor-associated macrophagic and lymphoplasmacytic inflammation (0: absent; 1, minimal; 2: mild; 3: moderate; 4: marked; 5: severe).

In this cohort, the immunohistochemical expression of ER, PR, HER2, Ki-67, EGFR, basal cytokeratins 5/6, basal cytokeratin 14, EGFR, AR, Bcl-2, FOXA1, and FoxP3 used as a regulatory T-cell marker has been previously described (19–21). For Sox2 immunohistochemistry, slides were heated at 95°C for 1 h in a basic buffer (CC1 cell conditioning medium, Roche Diagnostics 950-124) to achieve heat-induced epitope retrieval. Incubation with the primary antibody raised to Sox2 (clone SP76, rabbit monoclonal, Spring Bioscience M3760) was performed for 1 h at 37°C at 1:50 dilution in an antibody diluent (Roche Diagnostics 251-018). The detection system was the OptiView DAB IHC Detection Kit (Roche Diagnostics, 760-700), optimized for automated immunohistochemistry (Benchmark XT stainer, Ventana Medical Systems, Roche Diagnostics). Sox2 was quantified as an index, the percentage of positive neoplastic cells among at least 500 cancer cells. Further details on the immunohistochemical methods are provided in the Supplementary Material.

### Statistical Methods

Statistical analyses were conducted using the MedCalc® statistical software (Ostend, Belgium). The threshold for Sox2 positivity (>42%) was calculated according to receiver-operating characteristic (ROC) curves that best discriminated between cats that died due to their mammary carcinoma and cats that did not die from cancer within 2 years post-diagnosis.

Comparisons between Sox2-positive and Sox2-negative FMCs were done using Fisher's exact tests for categorical variables with two categories, chi-squared tests for categorical variables with more than two categories, or one-way analyses of variance for continuous variables.

Univariate survival analyses relied on the Kaplan-Meier method and log-rank tests, while Cox proportional-hazards models were used for multivariate survival analyses. The results are reported using the Hazard Ratio (HR), its 95% confidence interval (95% CI), and the *p*-value of each covariate. A *p*-value <0.05 was considered significant.

## Results

### Sox2 Immunohistochemistry

The immunohistochemical protocol for Sox2 detection gave a faint non-specific background staining in the cytoplasm of mast cells and in the cerebellar molecular layer. Otherwise, Sox2-specific signals were strictly nuclear. Sox2 expression was not detected at all in the normal mammary gland and hyperplasic mammary gland without atypia. Surrounding FMCs, occasional Sox2 expression was observed in mammary lobular or ductal atypical hyperplasia. A short description of Sox2 expression in other non-neoplastic feline tissues is presented in Supplementary Figure 1.

### Patient and Tumor Characteristics

The main patient and tumor characteristics are presented in Supplementary Table 1. Most cats (111/180, 62%) were intact females, and the mean age at diagnosis was 11.1 ± 2.7 years. Most cases (109/180, 61%) were diagnosed at stage III. The cribriform and solid histological subtypes predominated. According to the mitotic-modified Elston and Ellis grading system, most cases (101/180, 56%) were of grade II. Lymphovascular invasion was present in 109 cases (61%). The proportion of ER+ and PR+ cases varied considerably according to the threshold for positivity applied (≥1%, ≥10%, or >2 points in Allred score). A particularity of this cohort was the absence of HER2 scores 3+ by immunohistochemistry, related to the fact the protocol was optimized to avoid HER2 positive signals in the normal mammary gland, as recommended for breast cancers. According to the FMC immunophenotypes defined by Soares et al. most cases (141/180, 78%) were Luminal-B HER2–. However, according to the immunophenotypes inspired from breast cancers, most cases (113/180, 63%) were triple-negative basal-like.

### Sox2 Expression in FMCs and Clinical-Pathologic Associations

**Figure 1:**
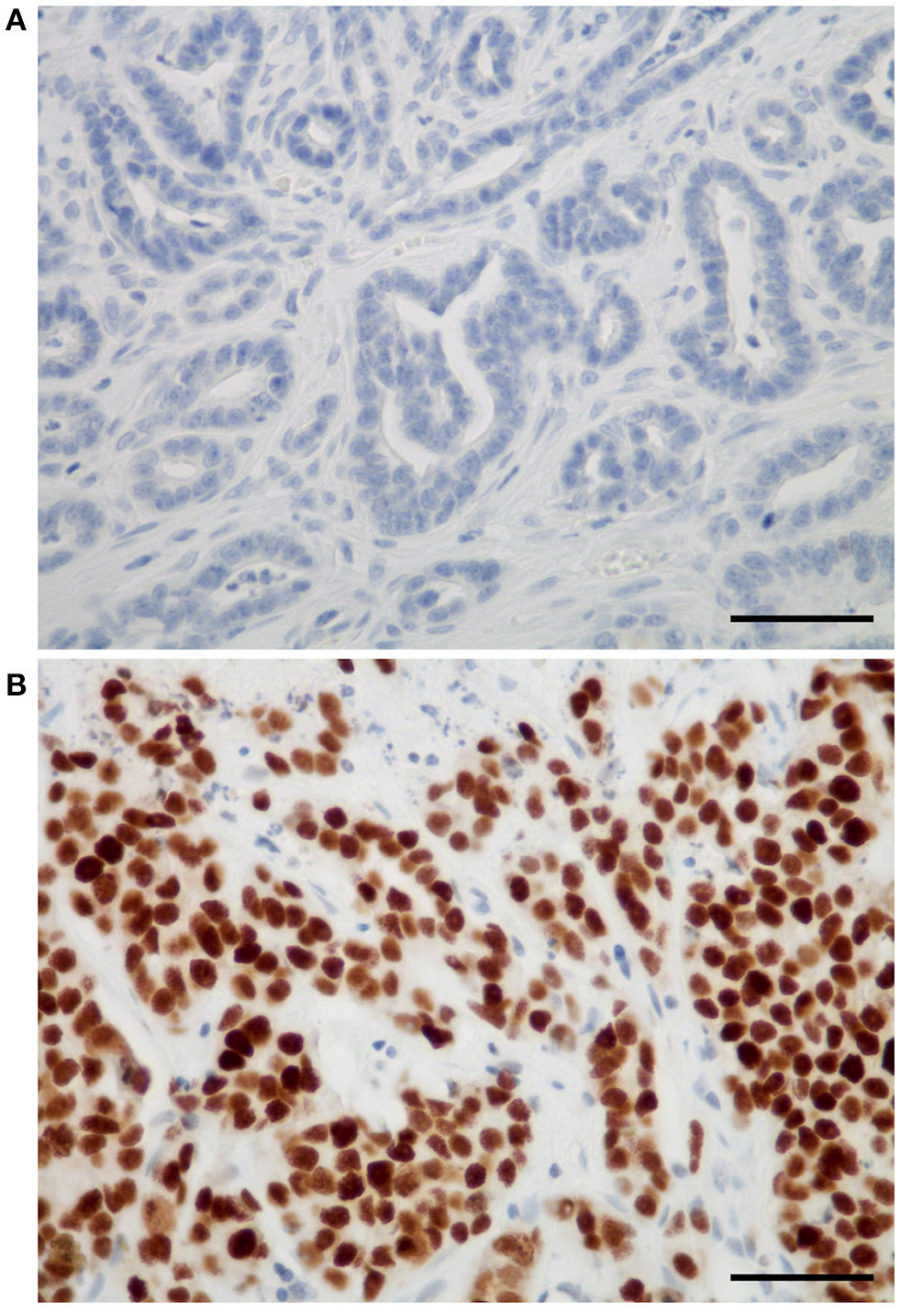
Sox2 expression in feline mammary carcinomas. **(A)** Example of a feline tubular mammary carcinoma without any Sox2 expression. **(B)** Example of a feline solid mammary carcinoma with very high Sox2 expression. The positive immunohistochemical signal was strictly nuclear, and restricted to neoplastic cells (absent in stromal cells). Sox2 immunohistochemistry, original magnification 400×, scale bars = 50 micrometers.

In FMCs, Sox2 expression was restricted to neoplastic cells, and not found in cancer-associated fibroblasts, tumor-infiltrating lymphocytes, endothelial cells, or any other stromal cell. The mean Sox2 index in FMCs was 38 ± 30% (median 33.5%, range 0–100%). Twenty-six (26) FMCs (14%) were totally devoid of Sox2 expression (Figure 1A), while eight FMCs (4%) expressed Sox2 in ≥90% of their neoplastic cells (Figure 1B). At threshold >42% for positivity, 79/180 FMCs (44%) were considered Sox2-positive.

Compared to Sox2-negative FMCs, Sox2-positive FMCs were diagnosed at a later age, were more likely to be associated with lymphovascular invasion and dermal infiltration, were more proliferative, and had a lower PR and FOXA1 expression (Table 1). However, Sox2 was not significantly associated with the neutering status, clinical and pathologic tumor size, clinical stages, histological types and histological grades, ER, basal markers, and the luminal or triple-negative phenotypes.

**Table 1:** Clinical-pathologic associations with Sox2 expression evaluated at >42% cutoff in feline mammary carcinomas (*N* = 180).

**Clinical-pathologic data**	**Sox2+ (*N* = 79)**	**Sox2– (*N* = 101)**	**Odds ratio (95% CI) for Sox2+ FMCs**	***P***
Age (years)	11.6 ± 2.7	10.7 ± 2.7	n/a	0.0360
Lymphovascular invasion (*N* = 109)	56 (71%)	53 (52%)	2.21 (1.19–4.12)	0.0186
No LVI (*N* = 71)	23 (29%)	48 (48%)		
Dermal infiltration (*N* = 113)	60 (76%)	53 (52%)	2.86 (1.50–5.46)	0.0021
No dermal infiltration (*N* = 67)	19 (24%)	48 (48%)		
Ki-67 ≥42% (*N* = 109)	55 (70%)	54 (53%)	1.99 (1.07–3.69)	0.0406
Ki-67 <42% (*N* = 71)	24 (30%)	47 (47%)		
PR index (%)	0.4 ± 1.4	4.9 ± 14.3	n/a	0.0060
PR ≥1% (*N* = 30) (a)	7 (9%)	23 (23%)	0.33 (0.13–0.82)	0.0224
PR <1% (*N* = 150)	72 (91%)	78 (77%)		
FOXA1 index (%)	1.1 ± 2.7	5.9 ± 12.6	n/a	0.0010
FOXA1 ≥1% (*N* = 64)	21 (27%)	43 (43%)	0.49 (0.26–0.93)	0.0387
FOXA1 <1% (*N* = 116)	58 (73%)	58 (57%)		

*(a) There was also a significant association between Sox2 at threshold >42% and PR at threshold ≥ 10% (p = 0.0025), however the 13 PR+ cases were all Sox2–, which prevented from calculating odds ratios*.

*n/a, not applicable*.

Expressed as a continuous variable (index), Sox2 expression showed positive associations with lymphovascular invasion, dermal infiltration, the Ki-67 proliferation index, and peritumoral regulatory T cells, while there were negative associations between Sox2 and PR and FOXA1 (Table 2).

**Table 2:** Clinical-pathologic associations with Sox2 expression expressed as an index in feline mammary carcinomas (*N* = 180).

**Clinical-pathologic data**		**Sox2 index (mean ± SD)**	***P***
Lymphovascular invasion	Yes (*N* = 109)	43 ± 30%	0.012
	No (*N* = 71)	31 ± 27%	
Dermal infiltration	Yes (*N* = 113)	44 ± 29%	0.001
	No (*N* = 67)	29 ± 28%	
Ki-67 (threshold ≥20%)	High ≥20% (*N* = 169)	40 ± 30%	0.013
	Low <20% (*N* = 11)	17 ± 17%	
Ki-67 (threshold ≥42%)	High ≥42% (*N* = 109)	44 ± 30%	0.001
	Low <42% (*N* = 71)	29 ± 28%	
PR (threshold ≥1%)	Positive ≥1% (*N* = 30)	27 ± 22%	0.028
	Negative <1% (*N* = 150)	40 ± 31%	
PR (threshold ≥10%)	Positive ≥10% (*N* = 13)	17 ± 13%	0.008
	Negative <10% (*N* = 167)	40 ± 30%	
FOXA1	Positive ≥1% (*N* = 64)	31 ± 26%	0.018
	Negative <1% (*N* = 116)	42 ± 31%	
Peritumoral regulatory T cells	≥575 /mm^2^ (*N* = 126)	42 ± 30%	0.017
	<575 /mm^2^ (*N* = 54)	30 ± 28%	

In the 57 luminal FMCs, Sox2 was associated with a larger tumor size, more advanced clinical stage, higher histological grades, squamous differentiation, lymphovascular invasion, dermal infiltration, moderate to severe tumor-associated inflammation, higher Ki-67 indexes, and increased numbers of intratumoral Tregs, but lower PR and FOXA1 expression (Supplementary Tables 2, 3). There were no significant associations between Sox2 and ER or the FMC immunophenotypes.

In the 123 triple-negative FMCs, Sox2 expression showed a positive association with the clinical tumor size, dermal infiltration, the Ki-67 proliferation index, AR expression, and intratumoral Treg numbers (Supplementary Tables 4, 5). There was also a non-significant trend toward a positive association between Sox2 positivity and the mucinous histological type (*p* = 0.051). There were no significant associations between Sox2 and basal markers (EGFR, cytokeratins 5/6, cytokeratin 14).

### Prognostic Significance of Sox2 Expression in FMCs

In the FMCs analyzed, positivity to Sox2 was associated with shorter DMFI, DFI, and cancer-specific survival, however there were no significant associations between Sox2 expression and the locoregional recurrence risk, or overall survival. An interesting finding was that Sox2 was complementary to AR in FMC prognostication.

#### Distant Metastasis-Free Interval

**Figure 2:**
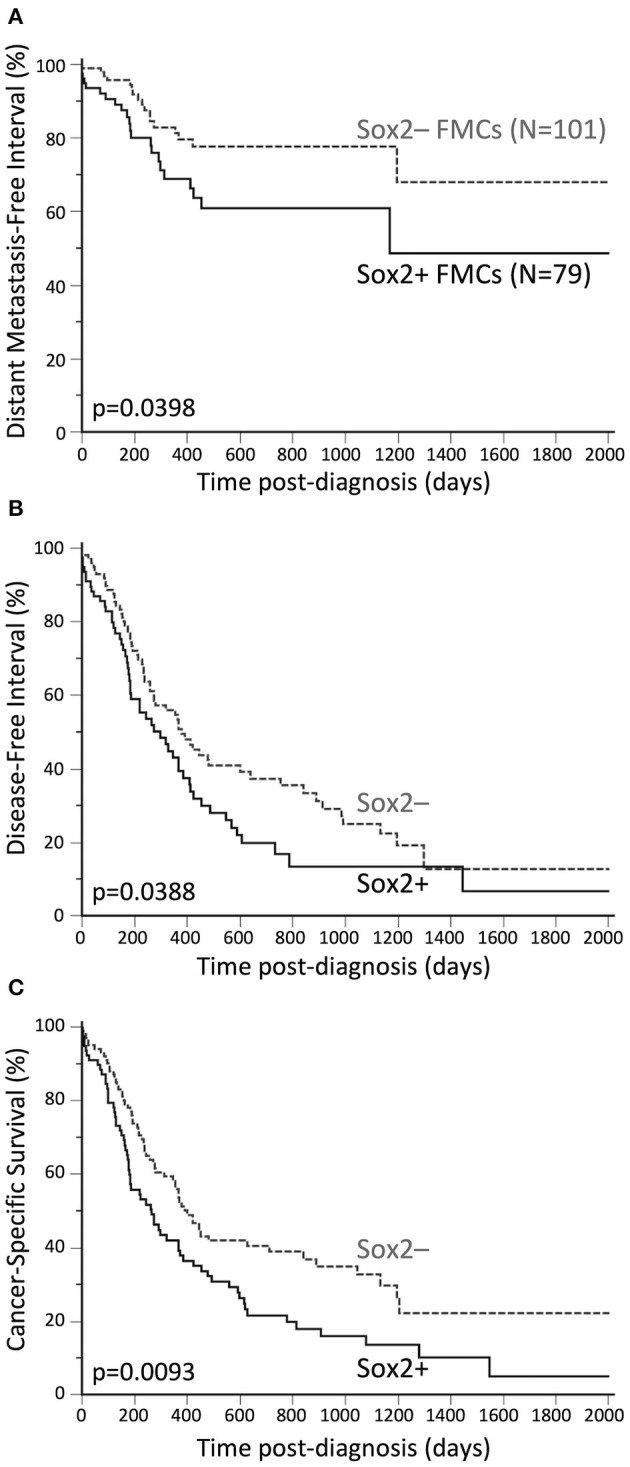
Unfavorable prognostic significance of Sox2 in FMCs. **(A)** Distant Metastasis-Free Interval. The probabilities of distant metastasis at 2 years post-diagnosis were 22% in the Sox2– group, and 39% in the Sox2+ group. **(B)** Disease-free interval. The median DFI was 12.4 months in the Sox2– group, and 9.7 months in the Sox2+ group. The probabilities of cancer progression at 2 years post diagnosis were 63% and 80% in the Sox2– and Sox2+ groups, respectively. **(C)** Cancer-specific survival. The median SS times were 13.1 and 8.7 months in the Sox2– and Sox2+ groups, respectively. At 2 years post-diagnosis, 61% of the cats in the Sox2– group, and 78% of the cats in the Sox2+ group had died from cancer. Kaplan-Meier curves.

Compared to Sox2-negative FMCs, Sox2-positive carcinomas were associated with an almost 2-fold increased risk of distant metastasis over time (HR = 1.87, 95% CI 1.00–3.51, *p* = 0.0398, Figure 2A). This was confirmed by multivariate analysis, as Sox2 was associated with higher probabilities of distant metastasis (HR = 2.01, 95% CI 1.08–3.75, *p* = 0.0288), independently of tumor size and AR expression (*p* = 0.0021, Cox proportional-hazard regression, Table 3).

**Table 3:** Unfavorable prognostic value of Sox2 (at >42% cutoff) in feline mammary carcinomas (multivariate survival analyses, *N* = 180 cats).

**DMFI (*P* = 0.0021)**	**Hazard ratio**	**95% CI**	***P***
Sox2+ vs. Sox2–	2.01	1.08–3.75	0.0288
Pathologic tumor size <20 vs. ≥20 mm	0.52	0.27–0.98	0.0428
AR index (continuous, in %)	0.98	0.97–0.99	0.0156
**Disease-free interval (*P* < 0.0001)**	**Hazard ratio**	**95% CI**	***P***
Sox2+ vs. Sox2–	1.48	1.02–2.15	0.0420
Pathologic tumor size <20 vs. ≥20 mm	0.61	0.42–0.89	0.0107
ER index (continuous, in %)	1.03	1.01–1.04	0.0002
AR+ vs. AR–	0.53	0.31–0.90	**0**.
**Specific survival (*P* < 0.0001)**	**Hazard ratio**	**95% CI**	***P***
Sox2+ vs. Sox2–	1.48	1.04–2.11	0.0292
Pathologic tumor size <20 vs. ≥20 mm	0.57	0.40–0.82	0.0025
Pathologic nodal stage pN0–NX vs. pN+	0.57	0.39–0.83	0.0035
Distant metastasis M1 vs. M0–MX	3.37	1.61–7.07	0.0013
AR+ vs. AR–	0.55	0.32–0.93	0.0265

#### Disease-Free Interval

By univariate analysis, Sox2 positivity of FMCs was associated with increased risk of cancer progression (HR = 1.47, 95% CI 1.00–2.15, *p* = 0.0388, Figure 2B). The probabilities of cancer progression at 1 year post diagnosis were 46% and 57% in the Sox2– and Sox2+ groups, respectively. The pejorative prognostic value of Sox2 was confirmed by multivariate survival analysis (HR = 1.48, 95% CI 1.02–2.15, *p* = 0.0420), with tumor size, ER and AR as the covariates (*p* < 0.0001, Cox proportional-hazard regression, Table 3). This model showed that Sox2 was complementary to hormone receptors ER and AR in predicting cancer progression, and also showed that ER and AR exerted opposite prognostic effects in FMCs, as increased ER expression was associated with cancer aggressiveness (recurrence, metastasis).

#### Cancer-Specific Survival

Sox2-positive FMCs were much more likely to kill feline patients than Sox2-negative carcinomas (HR = 1.57, 95% CI 1.10–2.25, *p* = 0.0093, Figure 2C). At 1 year post-diagnosis, 44% of the cats in the Sox2– group, and 58% of the cats in the Sox2+ group had died from cancer. By multivariate survival analysis, Sox2 was a robust pejorative prognostic factor (HR = 1.48, 95% CI 1.04–2.11, *p* = 0.0292), independent of cancer stage at diagnosis, and independent of AR, whose prognostic value was favorable (*p* < 0.0001 for the model, Table 3).

#### The Rare AR+Sox2– Phenotype Defined a Good-Prognosis Subgroup of FMCs

Because Sox2 and AR had independent (and opposite) prognostic effects in the FMCs of the present cohort, four FMC subgroups were analyzed according to AR and Sox2 expression: AR+Sox2+ (13/180, 7%), AR+Sox2– (19/180, 11%), AR–Sox2+ (66/180, 37%), and AR–Sox2– (82/180, 45%). Cox proportional-hazard models indicated however that the 3 subgroups AR+Sox2+, AR–Sox2+ and AR–Sox2– were not significantly associated with different patient outcomes (data not shown), while the AR+Sox2– phenotype was associated with excellent outcomes. This phenotype was found in 19/180 FMCs (11%), 9/57 luminal FMCs (16%), and 10/123 triple-negative FMCs (8%).

Association analyses revealed that AR+Sox2– FMCs were more likely to be node-negative, diagnosed at clinical stages I–II, and free of lymphovascular invasion compared to other FMCs (Supplementary Table 6). AR+Sox2– FMCs were also more likely to be of histological grades I–II according to the novel grading system for FMCs, which comprises lymphovascular invasion in its definition. The AR+Sox2– phenotype was also associated with a lower probability of dermal invasion and squamous differentiation and a lower proliferation index than other phenotypes, but with higher PR, FOXA1, and Bcl-2 expression. Finally, peritumoral Tregs were less numerous around AR+Sox2– FMCs than around other FMCs (Supplementary Table 6). There were no significant associations between the AR + Sox2– phenotype and patient age and breed, the neutering status, tumor size, multicentricity, histological types, the histological grades according to Elston and Ellis or the mitotic-modified system, ER and HER2 expression. The AR+Sox2– phenotype was not significantly associated with the immunophenotypes defined for FMCs (27), but was associated with those inspired by Nielsen et al. (28) and Cheang et al. (29) for breast cancers: there was a slight over-representation of Luminal-A cases among AR+Sox2– FMCs, while none of the triple-negative non-basal–like FMCs were AR+Sox2– (Supplementary Table 6).

**Figure 3:**
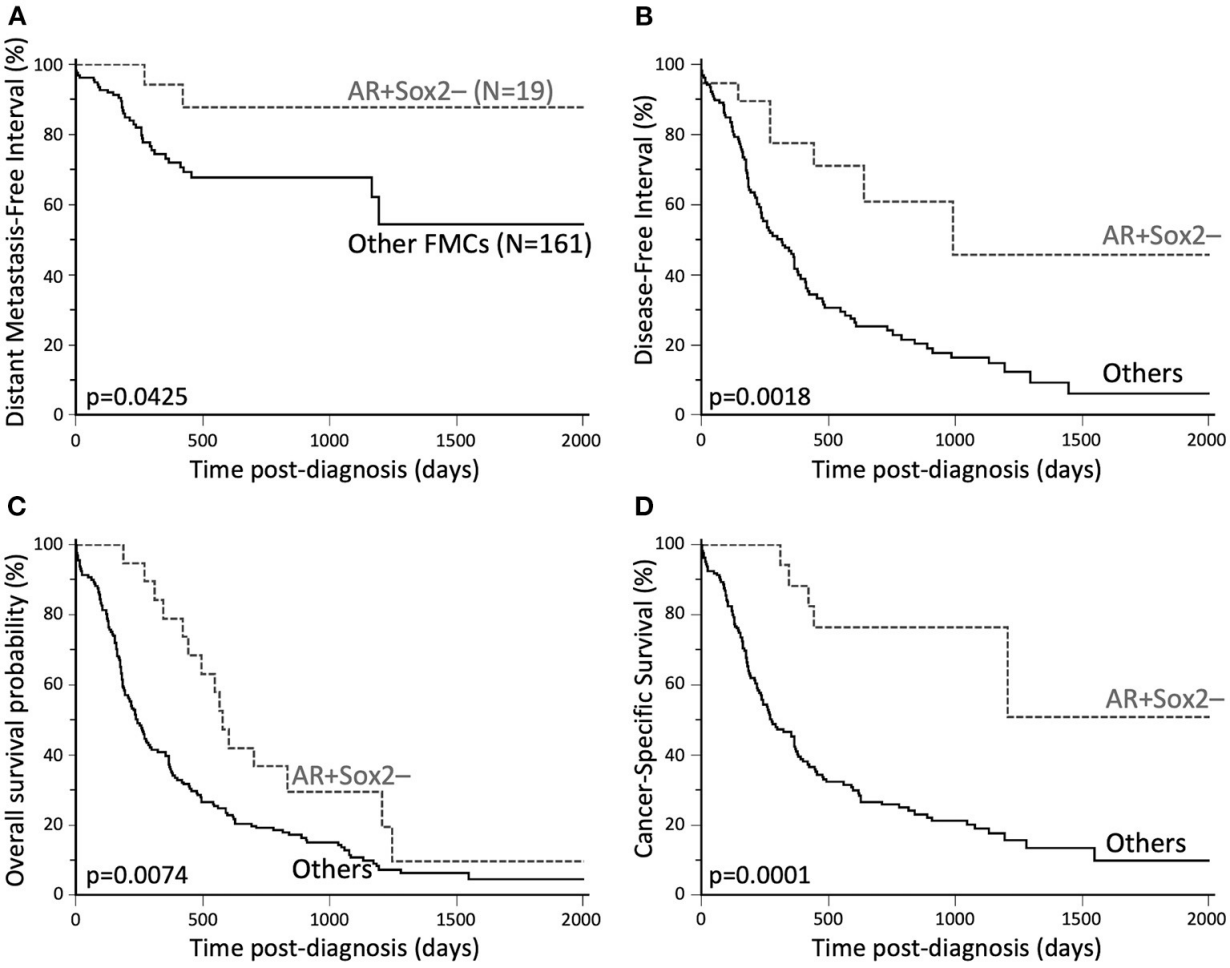
Favorable outcomes associated with AR+Sox2–FMCs. **(A)** Distant Metastasis-Free Interval. The probabilities of distant metastasis at 2 years post-diagnosis were 12% in the AR+Sox2– group, and 32% for other FMCs. **(B)** Disease-free interval. The median DFI was 32.5 months in the AR+Sox2– group, and 10.4 months for other FMCs. The probabilities of cancer progression at 2 years post diagnosis were 29% and 75% for AR+Sox2– and other FMCs, respectively. **(C)** Overall survival. The median OS times were 19.0 and 8.0 months in the AR+Sox2– and other-FMC groups, respectively. **(D)** Cancer-specific survival. At 2 years post-diagnosis, 23% of the cats in the AR+Sox2– group, and 74% of the cats with other FMCs had died from cancer. Kaplan-Meier curves.

In the entire cohort (*N* = 180), the AR + Sox2– phenotype was associated with decreased locoregional recurrence risk (HR = 0.36, 95% CI 0.22–0.62, *p* = 0.0056), improved DMFI (HR = 0.34, 95% CI 0.15–0.74, *p* = 0.0425, Figure 3A), improved DFI (HR = 0.35, 95% CI 0.21–0.56, *p* = 0.0018, Figure 3B), improved overall survival (HR = 0.51, 95% CI 0.34–0.76, *p* = 0.0074, Figure 3C), and decreased probabilities of cancer-related death (HR = 0.24, 95% CI 0.16–0.38, *p* = 0.0001, Figure 3D). Associations between AR+Sox2– and favorable outcomes were confirmed by multivariate survival analyses (Supplementary Table 7). The favorable prognostic value of the AR+Sox2– phenotype was also confirmed separately in luminal and triple-negative FMCs (Supplementary Material).

## Discussion

Feline and human Sox2 proteins share 98.8% identity in amino acid sequence (30), which facilitated Sox2 immunohistochemistry in cats using an anti-human Sox2 primary antibody. Sox2 was totally absent from the normal mammary gland, as reported in humans (31), but most of the FMCs analyzed harbored Sox2-positive tumor cells. Using a high threshold for positivity (Sox2 index >42%), 44% of the FMCs were Sox2-positive. By comparison, only 9–33% of breast cancers are Sox2-positive at threshold >0 (7, 9–13), and 10–19% at threshold ≥1% (14, 16). Thus, FMCs were characterized by much higher Sox2 expression than reported in breast cancers.

However, the clinical-pathologic associations with Sox2 expression were similar in both species. In both luminal and triple-negative FMCs, a positive association was found between Sox2 expression and tumor size. In breast cancers also, which are luminal in approximately 70% of the cases (32), Sox2 has been positively associated with tumor size in consecutive series (11–14) and a meta-analysis (15). This suggests that Sox2-positive mammary carcinomas may have a growth advantage over Sox2-negative ones, or that Sox2 expression is acquired or positively selected during local tumor growth.

In the FMCs analyzed, a positive association was found between Sox2 and lymphovascular invasion. By comparison in breast cancers, such associations are not significant (11, 14, 16), but Sox2 expression is associated with axillary lymph node metastasis (8, 9, 11, 15, 33), which is a step further than lymphovascular invasion.

In luminal FMCs, a positive association was observed between Sox2 expression and higher histological grades, in agreement with numerous reports in breast cancers (9, 14–16, 34). Moreover, Gwak et al. reported that Sox2 is especially associated with higher grade in hormone receptor-positive breast cancers, while not significantly in hormone receptor-negative carcinomas (16), as found in the present study on FMCs.

An association was found between Sox2 positivity and higher Ki-67 indexes in the entire cohort, as well as in luminal and triple-negative FMCs separately. This is in agreement with previous studies in breast cancers (13, 14, 16). A likely explanation is that Sox2 facilitates the G1/S transition of the cell cycle and up-regulates the *CCND1* (cyclin D1) gene, as demonstrated in the luminal MCF-7 and triple-negative MDA-MB-231 breast cancer cell lines (6).

This study did not demonstrate any significant Sox2/HER2 associations, as reported in human breast cancer (15). However, the 18 FMCs with a 2+ score by HER2 immunohistochemistry were not evaluated by dual-probe *in situ* hybridization, as recommended for breast cancers (35), so HER2-positive FMCs were underestimated in the present study. Recent studies indicate that *HER2* gene amplification can be expected in 4% of FMCs (36).

In the present study on FMCs, there were no significant associations between Sox2 and ER, in agreement with the meta-analysis by Zheng et al. in breast cancers (15). However, conflicting results exist in consecutive breast cancer series, with positive Sox2–ER associations (9), negative associations (12, 14), or no significant associations (10, 11, 16). In the FMCs examined, especially luminal ones, Sox2 positivity showed a negative association with PR and FOXA1. In breast cancers by comparison, the negative association between Sox2 and PR has been described by some authors (12, 14), but was not significant in a meta-analysis (15). The negative association between Sox2 and FOXA1 has been demonstrated in human breast and lung cancers, in which Sox2 represses *FOXA1* gene expression (37, 38). Considering that *PGR* (the PR-encoding gene) is a direct ER target gene (39), and FOXA1 is a critical pioneer factor for ER (40), it can be hypothesized that Sox2 altered ER transcriptional activity in FMCs of the present study as it does in breast cancers (41).

In triple-negative FMCs, Sox2 showed a positive association with AR expression. This is consistent with data obtained at the mRNA level using publicly available gene expression databases: *SOX2* and *AR* are positively correlated (*r* = 0.03, *p* = 0.0007) in breast cancers, although the correlation is not significant in triple-negative breast cancers in particular (*r* = 0.06, *p* = 0.0905) (42, 43). The results obtained in the present study indicated that Sox2 and hormone receptor associations differ in luminal and in triple-negative FMCs, with high Sox2 expression being associated with low PR expression in luminal FMCs, and high AR expression in triple-negative FMCs.

In both luminal and triple-negative FMCs, a positive association was found between Sox2 and intratumoral regulatory T cells. Interestingly in immune-competent mouse models of breast cancer, regulatory T cells enhance *SOX2* expression by mammary cancer cells in a paracrine manner, while Sox2-expressing tumor cells recruit Tregs through NF- κB/CCL1 [Nuclear Factor kappa B/Chemokine (C-C motif) ligand 1] signaling (44). Thus, Sox2-enriched mammary carcinomas may be prone to immune-suppressed tumor microenvironments. This could have therapeutic implications, as it suggests adding cancer immunotherapy to Sox2-targeted therapy for Sox2+ mammary cancers.

Overall, Sox2 associations with clinical-pathologic parameters tended to differ between luminal and triple-negative FMCs, with most associations found in luminal FMCs only (positive associations with the histological grade, lymphovascular invasion, and squamous differentiation, negative associations with PR and FOXA1). AR and Sox2 were positively associated in triple-negative FMCs only. These results suggest that luminal and triple-negative FMCs, defined in this study at ≥10% cutoff for ER and PR, are biologically distinct entities. Interestingly in human breast cancers, with cutoffs of ≥1% or ≥10% generally used for ER and PR (45), Sox2 associations also tend to differ between luminal and triple-negative breast cancers. Notably, the Sox2/histological grade association is significant in luminal but not triple-negative breast cancers (16).

Finally, the prognostic value of Sox2 expression was investigated in FMCs, and revealed that Sox2 positivity was associated with shorter distant metastasis-free interval, disease-free survival, and cancer-specific survival. In breast cancers, Sox2 is a weak prognostic factor, but has been associated with poor disease-free survival by univariate analyses (10, 13, 14, 33) and multivariate analyses, independently of the nodal stage, ER and HER2 (14) or independently of tumor size, nodal stage and PR (10), and poor overall survival, by univariate analyses only (13, 33). The rarely reported prognostic role of Sox2 in breast cancer patients seems contradictory with its major implications in mammary cancer cell biology (37), as Sox2 has been reported to increase breast cancer cell proliferation (6, 33, 46), favor invasion and metastasis (33, 46, 47), epithelial-to-mesenchymal transition, stemness and the Wnt-β-catenin signaling pathway (8, 44), and is associated with angiogenesis in breast cancer samples (48).

By multivariate survival analysis, mammary cancer progression (disease-free interval) in feline patients of the present study was independently modulated by Sox2, tumor size, ER and AR, with larger tumor size associated with poor prognosis, as frequently reported in FMCs (19–21, 49–55), AR associated with decreased probabilities of cancer progression (20), and ER expression associated with shortened DFI. This unfavorable effect of ER could seem paradoxical, as ER-positive breast cancers are associated with better outcomes than ER-negative breast cancers (56). However, *ESR1* (the ER-encoding gene) overexpression has been associated with worse relapse-free survival in ER-positive breast cancer patients (57), in line with the protumoral effects of ER signaling in mammary carcinomas (58). Indeed, ER-positive breast cancers usually respond well to endocrine therapy, which improves patient survival (59) despite ER being oncogenic in mammary carcinomas.

In this cohort, Sox2 and AR had independent and opposite effects on DMFI, DFI and cancer-specific survival, which triggered the analysis of FMC subgroups according to Sox2 and AR expression. The AR+Sox2– phenotype was associated with low probabilities of cancer-related death independently of tumor size, nodal and distant metastasis. Unfortunately for female cats with mammary carcinomas, this phenotype seems rare, encountered in only 11% of the cats of the present study. This rare AR+Sox2– phenotype was associated with favorable features, such as a negative nodal stage, an early clinical stage, and absence of lymphovascular invasion, low Ki-67 proliferation indexes, but high PR, FOXA1, and Bcl-2 expression. However, the AR+Sox2– phenotype was not specific of a given FMC immunophenotype, as it was found in some Luminal-A, Luminal-B, and triple-negative basal-like cases. To the authors' knowledge, AR and Sox2 have not been associated in breast cancer prognostic studies, though it could be interesting.

The main perspective following the present study would be to determine the mechanisms of Sox2 overexpression in FMCs. Sox2 overexpression may result from *SOX2* gene amplifications, however such amplifications are mainly reported in human serous ovarian cancers, lung squamous cell carcinomas, and glioblastomas, not in breast cancers (60, 61). In breast cancers, *SOX2* is up-regulated by the transcription factor FOXO1 (Forkhead box protein O1) (62), the long non-coding RNA SOX2OT (SOX2 overlapping transcript) (63), and the EGFR/Stat3 signaling pathway in a paracrine manner involving tumor-associated macrophages (64).

Unfortunately, a better understanding of Sox2 biology in mammary carcinomas is unlikely to have therapeutic implications in the near future. Sox2 is a poorly druggable transcription factor, but targeting signaling molecules upstream or downstream of Sox2 (Sox2 inducers and Sox2 targets, respectively) is under investigation in some human cancers (65).

In conclusion, we report here that the stem cell pluripotency factor Sox2 is commonly expressed in feline mammary carcinomas, is associated with higher proliferation, decreased PR and FOXA1, but higher AR expression, as well as poor outcomes. The unfavorable prognostic effect of Sox2 was complementary to the favorable effects of AR, and independent from cancer stage at diagnosis. Understanding the mechanisms underlying Sox2 overexpression would be a prerequisite before setting eventual Sox2-targeted clinical trials in feline mammary carcinoma patients.

## Data Availability Statement

The raw data supporting the conclusions of this article will be made available by the authors, without undue reservation.

## Ethics Statement

The animal study was reviewed and approved by CERVO, Comité d'Ethique en Recherche clinique et épidémiologique Vétérinaire d'Oniris, Oniris site Chantrerie, CS40706, 44307 Nantes cedex 3, France. Written informed consent was obtained from the owners for the participation of their animals in this study.

## Author Contributions

FN: conceptualization, project administration, supervision, writing, and original draft preparation. YT, ED, JA, and FN: format analysis, investigation, methodology, writing, review and editing. All authors contributed to the article and approved the submitted version.

## Conflict of Interest

The authors declare that the research was conducted in the absence of any commercial or financial relationships that could be construed as a potential conflict of interest.
