# Supplementary material for: Unfavorable Prognostic Effects of the Stem Cell Pluripotency Factor Sox2 in Feline Invasive Mammary Carcinomas
Source: Front Vet Sci. 2021 Jan 22;7:622019. doi: 10.3389/fvets.2020.622019 (PMC7862120; doi:10.3389/fvets.2020.622019)

Supplementary Material

# Supplementary Methods

**Immunohistochemistry (IHC)**

IHC was applied to detect:

- **p63**, a myoepithelial cell marker used to discriminate invasive mammary carcinomas (included) from mammary carcinomas in situ (excluded), using the murine primary antibody clone 4A4 (Abcam ab735, dilution 1:50, incubation time 32 minutes, revelation system iVIEW DAB IHC Detection kit)
- **LMO2** (LIM domain-only protein-2), a lymph endothelial cell marker used to confirm lymphovascular invasion when necessary (rabbit clone SP51, Spring M351, dilution 1:150, incubation time 1 hour and 32 minutes, revelation system OptiView DAB IHC Detection kit)
- **cytokeratins** to help visualizing nodal micrometastases and isolated tumor cells (murine clones AE1–AE3, Dako M3515, dilution 1:100, incubation time 44 minutes, revelation system iVIEW DAB IHC Detection kit)
- **ER alpha** (murine clone C311, Santa Cruz Biotechnology sc-787, dilution 1:50, incubation time 44 minutes, revelation system iVIEW DAB IHC Detection kit)
- **PR** (murine clone 10A9, Meridian Life Science K42546M, dilution 1:50, incubation time 1 hour, revelation system iVIEW DAB IHC Detection kit)
- **HER2** (rabbit clone 4B5, Roche Diagnostics 790-2991, prediluted, incubation time 8 minutes, revelation system UltraView universal DAB IHC Detection kit)
- the proliferation marker **Ki-67** (murine clone MIB1, Dako M7240, dilution 1:50, incubation time 32 minutes, revelation system iVIEW DAB IHC Detection)
- **Epidermal Growth Factor Receptor** (EGFR, rabbit clone 5B7, Roche Diagnostics 790-4347)
- **cytokeratins 5 and 6** (CK5/6, murine clone D5/16 B4, Dako M7237, dilution 1:50, incubation time 16 minutes, revelation system iVIEW DAB IHC Detection kit)
- **cytokeratin 14** (CK14, murine clone LL002, Santa Cruz Biotechnology sc-58724, dilution 1:100, incubation time 44 minutes, revelation system iVIEW DAB IHC Detection kit)
- **Bcl-2** (murine clone 7/Bcl-2, BD Transduction Laboratories 610539, dilution 1:50, incubation time 44 minutes, revelation system OptiView DAB IHC Detection kit)
- **AR** (rabbit clone SP107, Spring M4070, dilution 1:400, incubation time 1 hour and 20 minutes, revelation system OptiView DAB IHC Detection kit)
- **FOXA1** (rabbit clone SP88, Spring M3880, dilution 1:50, incubation time 1 hour and 20 minutes, revelation system OptiView DAB IHC Detection kit)
- and the Treg marker **Forkhead box protein P3** (FoxP3, rabbit clone SP97, Spring M3970, dilution 1:150, incubation time 32 minutes, revelation system OptiView DAB IHC Detection kit).

Most protocols required heat-induced antigen retrieval (HIER) in a basic buffer (CC1 cell conditioning medium, Roche Diagnostics 950-124) at 95°C, for 24 minutes (Bcl-2), 32 minutes (p63, CK5/6, FoxP3), 48 minutes (LMO2) or 1 hour (PR, HER2, Ki-67, EGFR, CK14). For pancytokeratin IHC (AE1–AE3), HIER for 8 minutes in the CC1 medium was followed by 8-minute pre-treatment with protease-1 (Ventana Medical Systems #760-2018). ER did not require any antigen retrieval.

Negative controls for IHC were included in each run, and consisted in replacing the primary antibody with normal rabbit or mouse serum (prediluted reagents, Roche Diagnostics, 760-1029 and 760-2014 respectively).

The positive internal controls were the mammary gland surrounding the carcinoma for ER, PR, CK5/6, CK14, AR, FOXA1, and Bcl-2; skin epidermis and hair follicles for Ki-67, CK5/6, and CK14; sebaceous glands for ER and AR; sweat glands for FOXA1; connective tissue cells surrounding hair follicles in the mid dermis and dermal papilla fibroblasts in the deep dermis for Sox2; peripheral nerves for HER2; some peritumoral lymphocytes for Bcl-2, FoxP3 and LMO2. For HER2, the pathway HER2 4-in-1 control slides (Roche Diagnostics) were used as external positive controls.

Three thresholds for positivity were used for ER and PR: ≥1% (commonly used in breast cancers) (45), ≥10% (cutoff with the best prognostic associations in FMCs), and >2 points in the Allred scoring system (used to determine FMC immunophenotypes according to Soares et al) (27). Briefly, Allred scores are generated by adding an intensity score (0 to 3 points: no staining, mild, moderate, intense staining) and a proportion score (0 to 5 points depending on the percentage of positive neoplastic cells at thresholds 1%, 10%, 33% and 66%). Three thresholds for positivity were used for Ki-67: ≥14% (according to Soares et al) (27), ≥20% (to discriminate between luminal-A and luminal-B cases as usually recommended for breast cancers), and ≥42% (best cutoff for prognostic analyses). HER2 was scored as recommended for human breast cancers (35), however in situ hybridization was not applied to cases with a 2+ score, due to unavailability of this method at our institution. The other cutoffs for positivity were estimated using receiver-operating characteristic (ROC) curve analyses: ≥1% for CK5/6, >10% for EGFR, ≥15% for CK14, ≥1% for FOXA1, and >65% for Bcl-2 (19). AR was considered positive for Allred scores 7 and 8 points, and negative for Allred scores 0 or 2–6 points (20).

Two systems for immunophenotype determination were applied. In the system proposed by Soares et al for FMCs, 6 immunophenotypes are defined: Luminal-A (ER and/or PR >2 points, HER2 scores 0 and 1+, Ki-67 <14%), Luminal-B HER2– (ER and/or PR >2 points, HER2 scores 0 and 1+, Ki-67 ≥14%), Luminal-B HER2+ (ER and/or PR >2 points, HER2 scores 2+ and 3+), HER2-positive (ER–, PR–, HER2 scores 2+ and 3+), triple-negative basal-like (ER and PR ≤2 points, HER2–, CK5/6+), and triple-negative normal-like (ER and PR ≤2 points, HER2–, CK5/6–) (27). In the system inspired by Nielsen et al and Cheang et al for breast cancers (28, 29), the mammary carcinomas were defined as luminal (ER+ and/or PR+) if they expressed ER and/or PR in ≥10% of tumor cells, and triple-negative (ER–, PR–, HER2–) if the ER and PR indexes were <10% and the HER2 score 0 to 2+. Luminal-A (Ki-67 <20%) and Luminal-B (Ki-67 ≥20%) were distinguished according to their proliferation indexes, while basal-like (CK5/6 ≥1% and/or EGFR >10%) and non-basal–like (CK5/6 <1% and EGFR ≤10%) triple-negative FMCs were distinguished according to basal markers.

# Supplementary Data

**Sox2 expression in normal feline tissues.**

Sox2 was intensely expressed in Malpighian (stratified squamous) epithelia of the oral cavity and esophagus (Supplementary Figure 1A), bronchial epithelium and glands (Supplementary Figure 1B), the gastric mucosa, connective tissue cells surrounding hair follicles in the mid dermis (Supplementary Figure 1C), dermal papilla fibroblasts of hair bulbs in the anagen phase of the hair follicle cycle (Supplementary Figure 1D), oocytes (Supplementary Figure 1E), and glial cells, including Bergmann glial cells (radial astrocytes) of the cerebellar cortex (Supplementary Figure 1F) and peripheral nerve Schwann cells.

An inconsistent to rare Sox2 expression was also observed in the adrenal medulla, pancreatic Langerhans islets, thyroid and parathyroid endocrine cells, the urinary bladder urothelium, salivary gland ducts, thymic epithelial cells, stromal cells of the ovarian cortex, testicular stromal cells, and smooth muscle cells of blood vessels and intestinal muscular layer.

Sox2 expression was not found in the epidermis, sebaceous glands, sweat glands of the skin, normal or hyperplastic mammary gland, small and large intestinal mucosa, oviduct, uterus, epididymis, kidney, liver, exocrine pancreas, cardiac and skeletal muscle, adipose tissue, and lymphoid cells.

**Prognostic value of the AR+Sox2– phenotype in luminal FMCs (N=57).**

In luminal FMCs, the AR+Sox2– phenotype was associated with improved DFI (HR = 0.41, 95% CI 0.21–0.80, *p* = 0.0246), improved overall survival (HR = 0.33, 95% CI 0.18–0.61, *p* = 0.0052), and decreased probabilities of cancer-related death (HR = 0.20, 95% CI 0.10–0.41, *p* = 0.0021).

However, the 9 AR+Sox2– luminal FMCs did not significantly differ from the other 48 luminal FMCs in terms of locoregional recurrence risk (*p* = 0.07) or distant metastasis-free interval (*p* = 0.10).

**Prognostic value of the AR+Sox2– phenotype in triple-negative FMCs (N=123).**

In triple-negative FMCs, the AR+Sox2– phenotype was associated with decreased locoregional recurrence risk (HR = 0.12, 95% CI 0.06–0.27, *p* = 0.0120), improved DFI (HR = 0.19, 95% CI 0.10–0.38, *p* = 0.0098), and decreased probabilities of cancer-related death (HR = 0.27, 95% CI 0.14–0.52, *p* = 0.0160).

However, the 10 AR+Sox2– triple-negative FMCs did not significantly differ from the other 113 triple-negative FMCs in terms of distant metastasis-free interval (*p* = 0.22) or overall survival (*p* = 0.32).

# Supplementary Tables

**Supplementary Table 1.** Patient and tumor characteristics at baseline (N=180).

| **Clinical-pathologic data** |  | **All FMCs (N=180)** |
| --- | --- | --- |
| **Age at diagnosis** | Years, mean ± SD | 11.1 ± 2.7 |
| **Neutering status** | Intact female | 111 (62%) |
|  | Neutered female | 69 (38%) |
| **Breed** | Domestic shorthair, Domestic longhair | 154 (86%) |
|  | Siamese | 15 (8%) |
|  | Persian | 3 (2%) |
|  | Other pure breeds | 8 (4%) |
| **History of contraception** | Yes | 76 (42%) |
|  | No | 18 (10%) |
|  | Unknown | 86 (48%) |
| **Location** | Mammary glands M1-M2 | 37 (21%) |
|  | Mammary glands M3-M4 | 105 (58%) |
|  | Multiple | 19 (10.5%) |
|  | Unknown | 19 (10.5%) |
| **Multifocality/Multicentricity** | Yes | 26 (14%) |
|  | Single FMC | 154 (86%) |
| **Type of surgery** | Lumpectomy | 9 (5%) |
|  | Single gland mastectomy | 27 (15%) |
|  | Regional mastectomy | 36 (20%) |
|  | Radical mastectomy | 108 (60%) |
| **Clinical tumor size** | T1 (<2 cm) | 67 (37%) |
|  | T2 (2–3 cm) | 77 (43%) |
|  | T3 (>3 cm) | 36 (20%) |

**Supplementary Table 1, continued.**

| **Clinical-pathologic data** |  | | **All FMCs (N=180)** |
| --- | --- | --- | --- |
| **Pathologic tumor size** | mm, mean ± SD (a) | 18.2 ± 7.1 | |
|  | pT1 <20 mm | 85 (47%) | |
|  | pT2 ≥20 mm | 95 (53%) | |
| **Pathologic nodal stage** | pN0 | 20 (11%) | |
|  | pNX | 59 (33%) | |
|  | pN+ | 101 (56%) | |
| **Distant metastasis** | M0 | 64 (36%) | |
|  | MX | 108 (60%) | |
|  | M1 | 8 (4%) | |
| **Clinical stage** | I | 34 (19%) | |
|  | II | 29 (16%) | |
|  | III | 109 (61%) | |
|  | IV | 8 (4%) | |
| **Histological type** | Adenosquamous | | 5 (3%) |
|  | Anaplastic | | 2 (1%) |
|  | Cribriform | | 84 (47%) |
|  | Mucinous | | 16 (9%) |
|  | Papillary | | 8 (4%) |
|  | Solid | | 40 (22%) |
|  | Squamous cell | | 1 (0.5%) |
|  | Tubular | | 10 (5.5%) |
|  | Tubulopapillary | | 14 (8%) |
| **Histological grade, EE** | I | | 9 (5%) |
|  | II | | 82 (46%) |
|  | III | | 89 (49%) |

(a) only available in 150 cases; 30 FMCs among the largest ones and/or FMCs with positive margins could not be precisely measured in mm on histological slides.

**Supplementary Table 1, continued.**

| **Clinical-pathologic data** |  | | **All FMCs (N=180)** |
| --- | --- | --- | --- |
| **Histological grade, MMEE (26)** | | I | 62 (34%) |
|  | | II | 101 (56%) |
|  | | III | 17 (9%) |
| **Histological grade, NGS (26)** | I | | 14 (8%) |
|  | II | | 59 (33%) |
|  | III | | 107 (59%) |
| **Lymphovascular invasion** | LVI+ | | 109 (61%) |
|  | LVI– | | 71 (39%) |
| **Dermal infiltration** | Yes | | 113 (63%) |
|  | No | | 67 (37%) |
| **Cutaneous ulceration** | Yes | | 37 (21%) |
|  | No | | 143 (79%) |
| **Muscle infiltration** | Yes | | 106 (59%) |
|  | No | | 74 (41%) |
| **Squamous differentiation** | Yes | | 83 (46%) |
|  | No | | 97 (54%) |
| **Central necrosis** | Yes | | 160 (89%) |
|  | No | | 20 (11%) |
| **Margin status** | Negative | | 83 (46%) |
|  | Positive | | 97 (54%) |
| **Tumor-associated inflammation** | Absent to mild | | 77 (43%) |
|  | Moderate to severe | | 103 (57%) |

**Supplementary Table 1, continued.**

| **Clinical-pathologic data** |  | **All FMCs (N=180)** |
| --- | --- | --- |
| **ER** | Index in %, mean ± SD | 10.0 ± 13.3 |
|  | ER ≥1% | 149 (83%) |
|  | ER ≥10% | 49 (27%) |
|  | ER >2 points (Allred score) | 157 (87%) |
| **PR** | Index in %, mean ± SD | 3.0 ± 11.0 |
|  | PR ≥1% | 30 (17%) |
|  | PR ≥10% | 13 (7%) |
|  | PR >2 points (Allred score) | 36 (20%) |
| **Ki-67** | Index in %, mean ± SD | 44.8 ± 14.0 |
|  | Ki-67 ≥14% | 177 (98%) |
|  | Ki-67 ≥20% | 169 (94%) |
|  | Ki-67 ≥42% | 109 (61%) |
| **HER2** | Score 0 | 103 (57%) |
|  | Score 1+ | 59 (33%) |
|  | Score 2+ | 18 (10%) |
|  | Score 3+ | 0 |
| **CK5/6** | Index in %, mean ± SD | 9.4 ± 15.9 |
|  | CK5/6 ≥1% | 113 (63%) |
| **CK14** | Index in %, mean ± SD | 33.7 ± 26.5 |
|  | CK14 ≥15% | 132 (73%) |
| **EGFR** | Index in %, mean ± SD | 52.9 ± 26.6 |
|  | EGFR >10% | 163 (91%) |
| **AR** | Index in %, mean ± SD | 45.0 ± 24.9 |
|  | AR ≥7 points (Allred score) | 32 (18%) |
| **Bcl-2** | Index in %, mean ± SD | 28.4 ± 30.4 |
|  | Bcl-2 >65% | 31 (17%) |

**Supplementary Table 1, end.**

| **Clinical-pathologic data** |  | | **All FMCs (N=180)** |
| --- | --- | --- | --- |
| **FOXA1** | | Index in %, mean ± SD | 3.8 ± 10.0 |
|  | | FOXA1 ≥1% | 64 (36%) |
| **Tregs, intratumoral stroma** | | /mm^2^, mean ± SD | 7 ± 9 |
| **Tregs, in contact with tumor cells** | | /mm^2^, mean ± SD | 6 ± 11 |
| **Tregs, peritumoral** | | /mm^2^, mean ± SD | 1064 ± 800 |
| **Immunophenotypes according to Soares et al (27)** | | Luminal-A | 2 (1%) |
|  |  | Luminal-B HER2– | 141 (78%) |
|  |  | Luminal B HER2+ | 17 (9.5%) |
|  | | HER2-positive | 1 (0.5%) |
|  | | Triple-Negative basal-like | 11 (6%) |
|  | | Triple-Negative normal-like | 8 (4%) |
| **Immunophenotypes inspired from breast cancers (28, 29)** | | Luminal-A | 8 (4%) |
|  |  | Luminal-B | 49 (27%) |
|  |  | Triple-negative basal-like | 113 (63%) |
|  |  | Triple-negative non-basal–like | 10 (6%) |

EE: Elston and Ellis histological grade

MMEE: Mitotic-Modified histological grade

NGS: Novel Grading System for FMCs

SD: standard deviation

Tregs: regulatory T cells

**Supplementary Table 2.** Clinical-pathologic associations with Sox2 expression evaluated at >42% cutoff in luminal mammary carcinomas (N=57).

| **Clinical-pathologic data** | **Sox2+ (N=22)** | **Sox2– (N=35)** | **Odds ratio (95% CI) for Sox2+ FMCs** | ***P*** |
| --- | --- | --- | --- | --- |
| **Pathologic tumor size (mm) (a)** | 20 ± 7 | 15 ± 6 | n/a | **0.0200** |
| **pT >18 mm** (N=24) | **14 (64%)** | 10 (29%) | **4.38 (1.41–13.65)** | **0.0196** |
| pT ≤18 mm (N=33) | 8 (36%) | 25 (71%) |  |  |
| **Clinical stage I–II** (N=23) | 5 (23%) | 18 (50%) | **0.28 (0.08–0.93)** | **0.0316** |
| Clinical stage III–IV (N=34) | **17 (77%)** | 17 (40%) |  |  |
| **Histological grade**, NGS I (N=6) | 1 (5%) | 5 (14%) | **0.17 (0.05–0.57)** (b) | **0.0127** |
| NGS grade II (N=21) | 4 (18%) | 17 (49%) |  |  |
| NGS grade III (N=30) | **17 (77%)** | 13 (37%) |  |  |
| **Squamous differentiation** (N=34) | **19 (86%)** | 15 (43%) | **8.44 (2.10–33.87)** | **0.0029** |
| Absent (N=23) | 3 (14%) | 20 (57%) |  |  |
| **Lymphovascular invasion** (N=32) | **19 (86%)** | 13 (37%) | **10.72 (2.65–43.36)** | **0.0007** |
| No LVI (N=25) | 3 (14%) | 22 (63%) |  |  |
| **Dermal infiltration** (N=33) | **18 (82%)** | 15 (43%) | **6.00 (1.68–21.44)** | **0.0087** |
| No dermal infiltration (N=24) | 4 (18%) | 20 (57%) |  |  |
| **Tumor-associated inflammation**, moderate to severe (N=34) | **18 (82%)** | 16 (46%) | **5.34 (1.50–19.04)** | **0.0152** |
| Absent to mild (N=23) | 4 (18%) | 19 (54%) |  |  |
| **Ki-67 index (%)** | **47 ± 12** | 38 ± 16 | n/a | **0.0320** |
| **PR index (%)** | 0.5 ± 1.6 | **13.7 ± 21.9** | n/a | **0.0070** |
| **PR ≥1%** (N=20) (c) | 2 (9%) | 18 (51%) | **0.09 (0.02–0.44)** | **0.0029** |
| PR <1% (N=37) | **20 (91%)** | 17 (49%) |  |  |
| **PR >2 points** (Allred score) (N=21) | 2 (9%) | 19 (54%) | **0.08 (0.02–0.40)** | **0.0016** |
| PR ≤2 points (N=36) | **20 (91%)** | 16 (46%) |  |  |
| **FOXA1 index (%)** | 1.4 ± 2.3 | **13.1 ± 18.3** | n/a | **0.0040** |

(a) Only available in 51 cases, because very large mammary carcinomas could not be fully included in paraffin blocks.

(b) Odds ratio calculated for NGS grades I–II versus NGS grade III.

(c) There was also a significant association between Sox2 at threshold >42% and PR at threshold ≥10% (*p* = 0.0034), however the 13 PR+ cases were all Sox2–, which prevented from calculating odds ratios.

n/a: not applicable

NGS: novel grading system according to Mills et al (26).

**Supplementary Table 3.** Clinical-pathologic associations with Sox2 expression expressed as an index in luminal mammary carcinomas (N=57).

| **Clinical-pathologic data** |  | **Sox2 index**  **(mean ± SD)** | ***P*** |
| --- | --- | --- | --- |
| **Histological grade, Elston and Ellis** | I (N=4) | 8 ± 7% | **0.045** |
|  | **II–III** (N=53) | **37 ± 28%** |  |
| **Histological grade, NGS** | NGS grade I (N=6) | 23 ± 27% | **0.026** |
|  | NGS grade II (N=21) | 25 ± 20% |  |
|  | **NGS grade III** (N=30) | **45 ± 31%** |  |
| **Lymphovascular invasion** | **Yes** (N=32) | **46 ± 30%** | **0.001** |
|  | No (N=25) | 22 ± 19% |  |
| **Dermal infiltration** | **Yes** (N=33) | **43 ± 30%** | **0.012** |
|  | No (N=24) | 25 ± 22% |  |
| **Squamous differentiation** | **Yes** (N=34) | **44 ± 29%** | **0.003** |
|  | No (N=23) | 22 ± 21% |  |
| **Tumor-associated** | Moderate to severe (N=34) | **43 ± 30%** | **0.013** |
| **inflammation** | Absent to mild (N=23) | 24 ± 22% |  |
| **Ki-67** (threshold ≥20%) | **High ≥20%** (N=49) | **39 ± 29%** | **0.031** |
|  | Low <20% (N=8) | 16 ± 9% |  |
| **Ki-67** (threshold ≥42%) | **High ≥42%** (N=26) | **46 ± 29%** | **0.002** |
|  | Low <42% (N=31) | 23 ± 22% |  |
| **PR** (threshold ≥1%) | Positive ≥1% (N=20) | 21 ± 17% | **0.004** |
|  | **Negative <1%** (N=37) | **43 ± 30%** |  |
| **PR** (threshold ≥10%) | Positive ≥10% (N=13) | 17 ± 13% | **0.008** |
|  | **Negative <10%** (N=44) | **41 ± 29%** |  |
| **PR** (threshold >2 points) | PR+ (>2 points) (N=21) | 22 ± 16% | **0.004** |
|  | **PR– (≤2 points)** (N=36) | **43 ± 31%** |  |
| **FOXA1** | Positive ≥1% (N=28) | 26 ± 22% | **0.011** |
|  | **Negative <1%** (N=29) | **45 ± 31%** |  |

**Supplementary Table 3, end.**

| **Clinical-pathologic data** |  | **Sox2 index**  **(mean ± SD)** | ***P*** |
| --- | --- | --- | --- |
| **Intratumoral Tregs in contact** | **≥2 /mm^2^** (N=31) | **43 ± 30%** | **0.022** |
| **with tumor cells** (a) | <2 /mm^2^ (N=26) | 26 ± 24% |  |
| **Tregs of the intratumoral** | **≥6 /mm^2^** (N=26) | **45 ± 32%** | **0.020** |
| **stroma** (a) | <6 /mm^2^ (N=31) | 27 ± 23% |  |

(a) In these analyses, intratumoral Tregs were counted separately if they were in contact with carcinomatous cells or if they were present in the intratumoral stroma.

NGS: novel grading system according to Mills et al (26).

**Supplementary Table 4.** Clinical-pathologic associations with Sox2 expression evaluated at >42% cutoff in triple-negative mammary carcinomas (N=123).

| **Clinical-pathologic data** | **Sox2+ (N=57)** | **Sox2– (N=66)** | **Odds ratio (95% CI) for Sox2+ FMCs** | ***P*** |
| --- | --- | --- | --- | --- |
| **Clinical tumor size** T1 (N=40) | 17 | **23** | **0.33 (0.14–0.78)** (a) | **0.0334** |
| T2 (N=53) | 20 | **33** |  |  |
| **T3** (N=30) | **20** | 10 |  |  |
| **AR index (%)** | **49 ± 23** | 39 ± 24 | n/a | **0.0230** |
| **Intratumoral Tregs in contact with tumor cells ≥2 /mm^2^** (N=50) | **29 (51%)** | 21 (32%) | **2.22 (1.07–4.62)** | **0.0498** |
| <2 /mm^2^ (N=73) | 28 (49%) | 45 (68%) |  |  |

n/a: not applicable

(a) Odds Ratio calculated for T1–T2 versus T3.

**Supplementary Table 5.** Clinical-pathologic associations with Sox2 expression expressed as an index in triple-negative mammary carcinomas (N=123).

| **Clinical-pathologic data** |  | **Sox2 index (mean ± SD)** | ***P*** |
| --- | --- | --- | --- |
| **Clinical tumor size** | T1 (N=40) | 38 ± 32% | **0.048** |
|  | T2 (N=53) | 34 ± 28% |  |
|  | **T3** (N=30) | **51 ± 30%** |  |
| **Dermal infiltration** | **Yes** (N=80) | **44 ± 29%** | **0.023** |
|  | No (N=43) | 31 ± 31% |  |
| **Ki-67** (threshold ≥42%) | **High ≥42%** (N=78) | **44 ± 30%** | **0.046** |
|  | Low <42% (N=45) | 32 ± 30% |  |

**Supplementary Table 6.** Clinical-pathologic features of AR+Sox2– FMCs (N = 19) compared to other FMCs (N = 161).

| **Clinical-pathologic data** | **AR+Sox2– (N=19)** | **Other FMCs (N=161)** | **Odds ratio (95% CI)** | ***P*** |
| --- | --- | --- | --- | --- |
| **Pathologic nodal stage pN1** (N=101) | 4 (21%) | **97 (60%)** | **0.18 (0.06–0.57)** | **0.0026** |
| pN0–pNX (N=79) | **15 (79%)** | 64 (40%) |  |  |
| **Clinical stage I** (N=34) | **6 (32%)** | 28 (17%) | **4.81 (1.73–13.38) (a)** | **0.0099** |
| Clinical stage II (N=29) | **7 (36%)** | 22 (14%) |  |  |
| Clinical stage III (N=109) | 6 (32%) | **103 (64%)** |  |  |
| Clinical stage IV (N=8) | 0 | **8 (5%)** |  |  |
| **Lymphovascular invasion** (N=109) | 3 (16%) | **106 (66%)** | **0.10 (0.03–0.36)** | **0.0001** |
| No LVI (N=71) | **16 (84%)** | 55 (34%) |  |  |
| **Histological grade, NGS** **I** (N=14) | **3 (16%)** | 11 (7%) | **9.73 (2.72–34.81) (b)** | **0.0002** |
| NGS grade II (N=59) | **13 (68%)** | 46 (29%) |  |  |
| NGS grade III (N=107) | 3 (16%) | **104 (64%)** |  |  |
| **Dermal infiltration** (N=113) | 6 (32%) | **107 (66%)** | **0.23 (0.08–0.64)** | **0.0065** |
| No dermal infiltration (N=67) | **13 (68%)** | 54 (34%) |  |  |
| **Squamous differentiation** (N=83) | 4 (21%) | **79 (49%)** | **0.28 (0.09–0.88)** | **0.0381** |
| Absent (N=97) | **15 (79%)** | 82 (51%) |  |  |
| **Ki-67 ≥20%** (N=169) | 15 (79%) | **154 (96%)** | **0.37 (0.11–1.26)** | **0.0179** |
| Ki-67 <20% (N=11) | **4 (21%)** | 7 (4%) |  |  |
| **PR index (%)** | **10.1 ± 13.2** | 2.1 ± 10.4 | n/a | **0.0020** |
| **PR ≥1%** (N=30) | **9 (47%)** | 21 (13%) | **6.00 (2.18–16.48)** | **0.0005** |
| PR <1% (N=150) | 10 (53%) | **140 (87%)** |  |  |
| **PR ≥10%** (N=13) | **7 (37%)** | 6 (4%) | **15.07 (4.37–52.00)** | **<0.0001** |
| PR <10% (N=167) | 12 (63%) | **155 (96%)** |  |  |

**Supplementary Table 6, end.**

| **Clinical-pathologic data** | **AR+Sox2– (N=19)** | **Other FMCs (N=161)** | **Odds ratio (95% CI)** | ***P*** |
| --- | --- | --- | --- | --- |
| **PR ≥3 points (Allred score)** (N=36) | **9 (47%)** | 27 (17%) | **4.47 (1.66–12.04)** | **0.0044** |
| PR ≤2 points (N=144) | 10 (53%) | **134 (83%)** |  |  |
| **FOXA1 index (%)** | **13.4 ± 15.7** | 2.7 ± 8.3 | n/a | **<0.001** |
| **Bcl-2 index (%)** | **49.4 ± 30.5** | 26.0 ± 29.5 | n/a | **0.0010** |
| **Bcl-2 ≥65%** (N=31) | **8 (42%)** | 23 (14%) | **4.36 (1.58–12.00)** | **0.0066** |
| Bcl-2 <65% (N=149) | 11 (58%) | **138 (86%)** |  |  |
| **Peritumoral Tregs /mm^2^** | 637 ± 515 | 1165 ± 814 | n/a | **0.0140** |
| **Peritumoral Tregs ≥575 /mm^2^** (N=126) | 8 (42%) | **118 (73%)** | **0.27 (0.10–0.72)** | **0.0111** |
| <575 /mm^2^ (N=54) | **11 (58%)** | 43 (27%) |  |  |
| **Immunophenotypes inspired from breast cancers** |  |  | n/a | **0.0497** |
| Luminal-A (N=8) | **3 (16%)** | 5 (3%) |  |  |
| Luminal-B (N=49) | 6 (32%) | 43 (27%) |  |  |
| Triple-negative basal-like (N=113) | 10 (52%) | 103 (64%) |  |  |
| Triple-negative non-basal–like (N=10) | 0 | **10 (6%)** |  |  |

(a) Odds ratio calculated for clinical stages I–II versus clinical stages III–IV.

(b) Odds ratio calculated for grades I–II versus grade III.

NGS: Novel Grading System according to Mills et al (26).

Tregs: regulatory T cells.

**Supplementary Table 7.** Favorable outcomes associated with the AR+ Sox2– phenotype (multivariate survival analyses, N = 180 FMCs).

| **Disease-Free Interval (*P* = 0.0001)** | **Hazard Ratio** | **95% CI** | ***P*** |
| --- | --- | --- | --- |
| **AR+Sox2–** versus Others | 0.36 | 0.17–0.79 | **0.0109** |
| **Clinical stage** III–IV versus I–II | 1.60 | 1.08–2.38 | **0.0210** |
| **Overall survival (*P* < 0.0001)** | **Hazard Ratio** | **95% CI** | ***P*** |
| **AR+Sox2–** versus Others | 0.58 | 0.34–0.99 | **0.0467** |
| **Pathologic tumor size** ≥20 versus <20 mm | 1.59 | 1.16–2.17 | **0.0038** |
| **Nodal stage** pN+ versus pN0–NX | 1.62 | 1.17–2.23 | **0.0035** |
| **Specific survival (*P* < 0.0001)** | **Hazard Ratio** | **95% CI** | ***P*** |
| **AR+Sox2–** versus Others | 0.26 | 0.11–0.65 | **0.0038** |
| **Pathologic tumor size** ≥20 versus <20 mm | 1.66 | 1.16–2.38 | **0.0057** |
| **Nodal stage** pN+ versus pN0–NX | 1.76 | 1.21–2.55 | **0.0032** |
| **Distant metastasis** M1 versus M0–MX | 3.24 | 1.55–6.76 | **0.0018** |

**Supplementary Figure 1.** Sox2 expression in normal feline tissues. The Sox2 protein was only observed in nuclear location. (A) Esophagus. Malpighian epithelial cells of the esophageal mucosa strongly expressed Sox2. (B) Lung. Bronchial epithelium and glands showed an intense positive signal. (C) Skin, dermis. Sox2 was expressed by some perifollicular connective tissue cells. (D) Skin, hair follicle. Intense nuclear Sox2 expression in dermal papilla fibroblasts. (E) Ovary, cortex. Intense Sox2 expression in oocytes. (F) Cerebellar cortex. Sox2 was expressed by Bergmann glial cells of the Purkinje layer. Sox2 immunohistochemistry, original magnification 400x, scale bars = 50 micrometers.


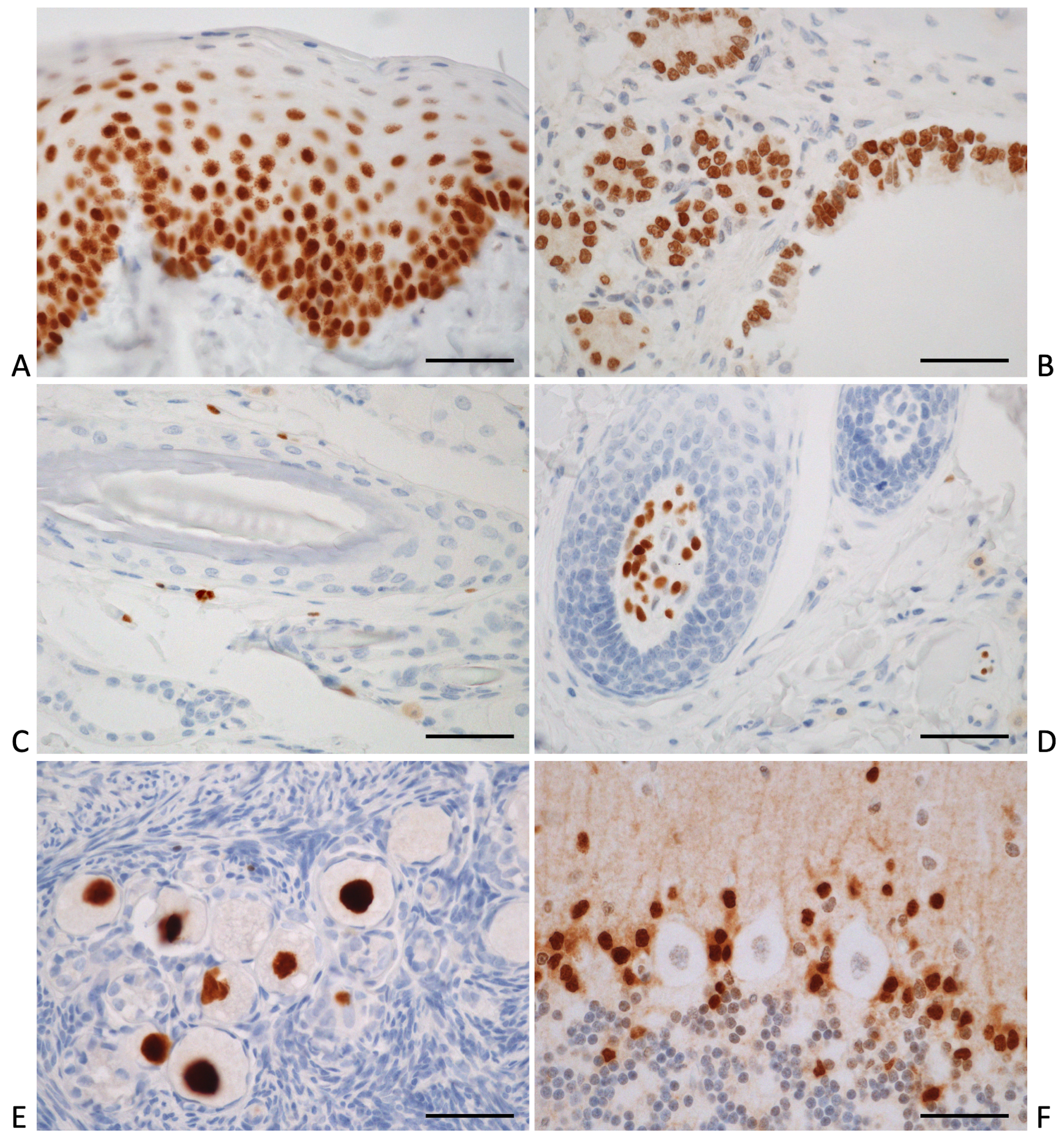

Supplement: Supplementary file 1 [file Table_1.DOCX]
